# Intranasally Administered MSC-Derived Extracellular Vesicles Reverse Cisplatin-Induced Cognitive Impairment

**DOI:** 10.3390/ijms241411862

**Published:** 2023-07-24

**Authors:** Bojana Milutinovic, Rajasekaran Mahalingam, Mayela Mendt, Luis Arroyo, Alexandre Seua, Shruti Dharmaraj, Elizabeth Shpall, Cobi J. Heijnen

**Affiliations:** 1 Laboratories of Neuroimmunology, Department of Symptom Research, Division of Internal Medicine, The University of Texas MD Anderson Cancer Center, Houston, TX 77030, USA; bmilutinovic@mdanderson.org (B.M.); rmahalingam@mdanderson.org (R.M.); luisda@utexas.edu (L.A.); aseua95@gmail.com (A.S.); shruti.dharmaraj@umaryland.edu (S.D.); 2 Department of Stem Cell Transplantation and Cellular Therapy, Division of Cancer Medicine, The University of Texas MD Anderson Cancer Center, Houston, TX 77030, USA; mcmendt@mdanderson.org (M.M.); eshpall@mdanderson.org (E.S.)

**Keywords:** chemobrain, cognition, MSC, extracellular vesicle, white matter, synaptic integrity, mitochondria

## Abstract

Neurotoxic side effects of chemotherapy include deficits in attention, memory, and executive functioning. Currently, there are no FDA-approved therapies. In mice, cisplatin causes long-term cognitive deficits, white matter damage, mitochondrial dysfunction, and loss of synaptic integrity. We hypothesized that MSC-derived small extracellular vesicles (sEVs) could restore cisplatin-induced cognitive impairments and brain damage. Animals were injected with cisplatin intraperitoneally and treated with MSC-derived sEVs intranasally 48 and 96 h after the last cisplatin injection. The puzzle box test (PBT) and the novel object place recognition test (NOPRT) were used to determine cognitive deficits. Synaptosomal mitochondrial morphology was analyzed by transmission electron microscopy. Immunohistochemistry using antibodies against synaptophysin and PSD95 was applied to assess synaptic loss. Black-Gold II staining was used to quantify white matter integrity. Our data show that sEVs enter the brain in 30 min and reverse the cisplatin-induced deficits in executive functioning and working and spatial memory. Abnormalities in mitochondrial morphology, loss of white matter, and synaptic integrity in the hippocampus were restored as well. Transcriptomic analysis revealed upregulation of regenerative functions after treatment with sEVs, pointing to a possible role of axonal guidance signaling, netrin signaling, and Wnt/Ca^2+^ signaling in recovery. Our data suggest that intranasal sEV treatment could become a novel therapeutic approach for the treatment of chemobrain.

## 1. Introduction

Chemotherapy-induced cognitive impairment (CICI), also known as chemobrain or chemofog, refers to a collection of symptoms experienced by patients treated for cancer with chemotherapies, including cisplatin. Chemobrain presents itself with problems with memory, attention and concentration, processing speed, executive function, and psychomotor skills. In 25% of patients, these symptoms can persist for years after treatment. The reported incidence varies from 30% in patients treated for childhood cancer [1] to 75% in breast cancer patients undergoing treatment [2,3] and has also been observed in patients treated for ovarian, lung, testicular, and head and neck cancer [4,5,6]. By 2030, the number of cancer survivors is projected to reach 23 million people in the US alone, and thus CICI will likely represent a growing public health concern [7]. Currently, there is no FDA-approved treatment for CICI.

Cisplatin is a platinum-based compound used for the treatment of ovarian, testicular, and bladder cancers [8]. Cisplatin can cross the blood-brain barrier to some extent, and doses detected in the brain are sufficient to damage hippocampal neurons in vitro [9]. To investigate the effect of cisplatin on cognition in order to identify potential therapeutic interventions, we developed a mouse model using a cumulative cisplatin dose of 23 mg/kg. A single cisplatin dose of 0.23 mg/kg corresponds to a human equivalent dose of 70 mg/m^2^, which is within the therapeutic range used for solid tumors [10,11]. In our mouse model, cisplatin induces long-lasting deficits in executive function, as well as in working and spatial memory [12,13,14,15,16,17,18,19]. Cisplatin treatment leads to mitochondrial damage in many tissues including the brain [17] by inducing mitochondrial accumulation of the tumor suppressor protein p53 [16,19,20,21]. We showed earlier that treatment with a p53 inhibitor pifithrin-μ prevents development of mitochondrial damage and cognitive deficits in mice [17]. Cisplatin also reduces myelin density and complexity in the cingulate and sensorimotor cortex [14,17] and compromises synaptic integrity in the hippocampus [13,15].

We have previously demonstrated that intranasal administration of human or murine bone marrow-derived MSCs leads to complete reversal of cognitive impairment and recovery of structural changes in cisplatin-treated mice [12,22]. To date, 1355 MSC-based clinical trials, completed or ongoing, are listed in the database of the US National Library of Medicine. These trials include, but are not limited to, diseases of the musculoskeletal system, CNS, lung and respiratory organs, and the immune and vascular systems. Therapeutic/clinical application of MSCs may include the risk of transferring mutated or damaged DNA [23]. Moreover, due to the relatively large size of MSCs, MSCs can be easily trapped in pulmonary capillaries after intravenous injections, the so-called pulmonary first-pass effect [24,25]. Recently, we also performed a feasibility and safety study of intranasal administration after perinatal ischemic stroke in neonates and concluded that no serious adverse events were observed [26].

It is now widely accepted that MSC secrete paracrine factors, including sEVs, which can confer part of their therapeutic properties [27,28]. Recent advances suggest that the MSC-derived vesicles may present a promising cell-free therapeutic alternative [29]. Notably, the small size of sEVs enables circulation through the capillaries, which potentially increases the effective “dose” reaching the target tissue. sEVs can be produced in compliance with GMP standards and formulated as an “off-the-shelf” therapeutic [30]. Moreover, assessment of sEVs in preclinical disease models shows that they are not immunogenic, which makes them attractive candidates for therapeutic application [23,30,31,32]. However, the majority of intravenously administered sEVs are taken up in the liver and the lungs by the reticuloendothelial cells shortly after injection [33]. Intranasal administration of EVs represents an efficacious strategy that circumvents this problem and facilitates efficient delivery of extracellular vesicles to the brain [34].

Here we tested the hypothesis that intranasal administration of MSC-derived sEVs restores cognition and repairs structural damage in the brains of cisplatin-treated mice. We investigated the effect of intranasal sEV treatment on executive function, as well as spatial and working memory of cisplatin-treated animals. We also monitored the distribution of sEV in the brain and meningeal compartment after intranasal administration. As a mechanism, we examined the potential restorative effect of sEVs on synaptic mitochondrial morphology, white matter organization, and synaptic integrity in the hippocampus. We also performed RNA sequencing of the hippocampus to explore the regenerative effects of sEVs to get more insight into the pathways activated by sEV treatment.

## 2. Results

### 2.1. Characterization of hBM MSC-Derived EVs

**Figure 1:**
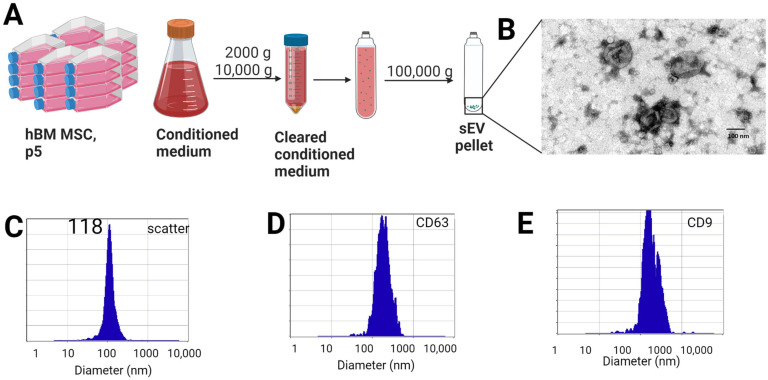
Isolation and characterization of hBM MSC sEVs. (**A**) Schematic representation of isolation of sEVs from passage 5 hBM MSC by differential centrifugation. (**B**) TEM analysis of sEVs reveals membrane-enveloped vesicles with typical cup-shaped morphology. Size bar—100 nm. (**C**) ZetaView analysis of a representative sample shows that the mean diameter of particles is 118 nm. Expression of typical markers of endosomal origin—CD63, CD9—was confirmed (**D**,**E**).

We prepared small extracellular vesicles from cell culture media of human bone marrow-derived (hBM) MSCs by differential centrifugation (Figure 1A). TEM assessment confirmed that the vesicles are membrane-enveloped with the cup-shaped morphology typical of exosomes (Figure 1B). ZetaView measurement showed that sEVs are homogenous in size, with a mean diameter of 118 nm (Std = 39.6 nm, x90 = 161.3 nm) (Figure 1C). The isolated sEVs expressed CD63 and CD9, which represent markers of endosomal origin (Figure 1D,E). The preparation was negative for the Golgi complex marker, GM130 (Supplementary Figure S1). Two independent preparations of sEVs showed a comparable biomarker profile (Supplementary Figure S1).

### 2.2. Intranasally Administered sEVs Restore Cognition in Cisplatin-Treated Mice

**Figure 2:**
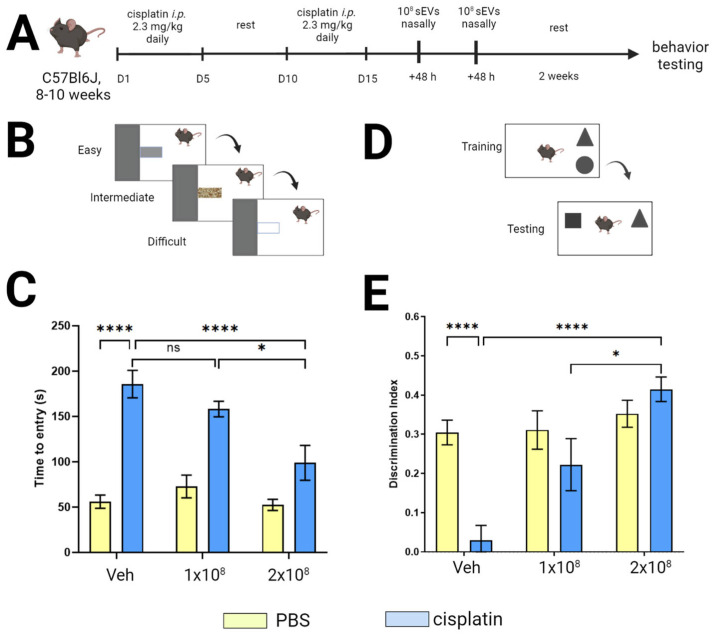
Intranasal administration of sEVs restores cognition in cisplatin-treated mice. (**A**) Timeline of the experiment. (**B**) Experimental setup of the three-phase PBT. (**C**) Average time to entry into the dark chamber recorded in four difficult trials (*n* = 12 M/8F). (**D**) NOPRT setup including training phase with familiar objects (triangle, circle) and testing phase with the novel object (square). (**E**) Average discrimination index (DI) during a 5-min testing window (*n* = 12M/8F). DI was calculated based on the formula (Tnovel − Tfamiliar)/(Tnovel + Tfamiliar). Data were analyzed with three-way ANOVA, for cisplatin, sEVs, and sex with Tukey’s post-hoc analysis, and the results are expressed as mean ± SEM. * *p* < 0.05, **** *p* < 0.0001, ns—not significant.

To examine the capacity of intranasally administered sEVs to reverse cisplatin-induced cognitive damage and functional and structural changes in the brain, we treated male and female C57BL/6 J mice with cisplatin (two cycles of five daily injections of 2.3 mg/kg in PBS with a 5-day rest in between; Figure 2A). sEVs (0.05 billion sEVs/3 µL in each nostril; total sEV dose 0.1 billion) were administered intranasally at 48 h (single dose) after the last cisplatin injection or in a separate cohort of mice at 48 and 96 h (double dose: 2 × 0.1 billion sEVs) after the last cisplatin injection. We used this treatment schedule because we have shown before that intranasal administration of MSCs at these timepoints reversed cisplatin-induced cognitive impairment [22]. Behavioral testing to assess cognitive function was started two weeks after the last dose of sEVs.

We assessed executive functioning using the puzzle box test (PBT). In this test, each mouse is placed individually in a brightly lit arena connected to a dark goal box by a tunnel. Mice prefer the dark, and when placed in the light chamber, they will escape to the dark goal box (Figure 2B). The test consists of three phases with increasing difficulty: open tunnel (easy trial); tunnel obstructed with bedding (intermediate trial); and a solid plug of the same color as the floor (difficult trial). An increase in time to reach the goal box during intermediate or difficult trials is a sign of impaired executive functioning. Consistent with our previous studies, cisplatin did not affect performance in the easy or intermediate trials (Supplementary Figure S2A,B). However, time to entry in the difficult trials was increased in cisplatin-treated mice, both males and females (Figure 2C). While administration of a single dose of sEVs did not improve performance of cisplatin-treated animals, intranasal administration of two doses of sEVs at 48 and 96 h after cisplatin treatment of male and female mice normalized performance in the PBT. These findings indicate that intranasal administration of two doses of sEV is sufficient to reverse the cisplatin-induced deficits in executive functioning. We did not detect any effects of sex, and there was no significant effect of sEV administration on behavior in control PBS-treated mice (Supplementary Figure S2C,D).

To test the effect of cisplatin and sEVs on spatial and working memory, we used the novel object place recognition test (NOPRT) (Figure 2D). This test uses the innate preference of mice for novelty. In rodents, the working memory is engaged when assessing sizes and shapes of objects, and spatial memory is engaged to assess the relative position of the object. During the training phase, each mouse is individually placed in an arena with two objects for 5 min, after which it is returned to its home cage for 60 min. During the test phase, one of the objects is replaced by a novel object in a novel location. Healthy mice will predominantly explore the novel object, resulting in a discrimination index > 0. Cisplatin treatment significantly reduced the preference for the novel object (reduced discrimination index) in both males and females (Figure 2C). Like in the PBT, only administration of two sEV doses reversed the negative effect of cisplatin and caused the discrimination index to increase to a level comparable with that of PBS-treated mice (Figure 2C). There was no significant difference in the performance of these tests between males and females (Supplementary Figure S3). The total interaction time, i.e., the total time spent exploring both objects during the test phase, did not differ between cisplatin and PBS-treated mice, indicating that differences in the discrimination index cannot be attributed to a lack of motivation to perform.

These results show that cisplatin-induced cognitive impairments are reversed by intranasal administration of two doses of sEVs, resulting in fully restored executive functioning and in restoration of spatial and working memory. Intranasal administration of a single dose of sEV did not significantly improve performance of cisplatin-treated animals in the cognitive assessment tests mentioned above.

### 2.3. Tracking of Nasally Administered sEV in the Brain

To examine the spatio-temporal dynamics of intranasally administered sEVs, we labelled the vesicles with the fluorescent dye DiD (1,1′-Dioctadecyl-3,3,3′,3′-Tetramethylindodicarbocyanine). Vesicle morphology was preserved after staining, and no dye aggregates were detected by TEM (Supplementary Figure S4).

**Figure 3:**
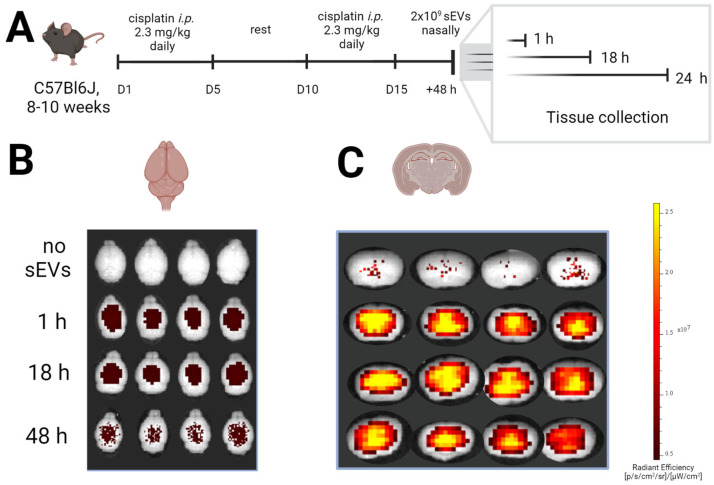
Spatiotemporal distribution of DiD-labelled sEVs in the brains of cisplatin mice. (**A**) Timeline of the experiment. (**B**) Representative images of fluorescent signal in the brain recorded by IVIS Lumina, dorsal view. (**C**) Representative images of fluorescent signal in the brain recorded by IVIS Lumina, coronal view. The brains were sliced in the level of the hippocampus (−1.28 mm from bregma) and imaged.

Mice were treated with cisplatin, and DiD-labeled sEVs were administered intranasally 48 h after the last dose of cisplatin (Figure 3A). IVIS imaging detected a fluorescent signal in the brain one hour after treatment. The signal was still detected at 18 h after sEV dosing with the same intensity then observed after one hour (Figure 3B). The intensity of the DiD signal decreased slightly at 48h after treatment, but this change was not statistically significant (Supplementary Figure S5). When the brains were imaged in a coronal plane −1.28 mm from the bregma (area containing the hippocampus), an intense signal was detected in the center, indicating that the intranasally administered sEV distributed into the brain parenchyma (Figure 3C).

### 2.4. Intranasal Administration of sEVs Reverse Cisplatin-Induced White Matter Damage

**Figure 4:**
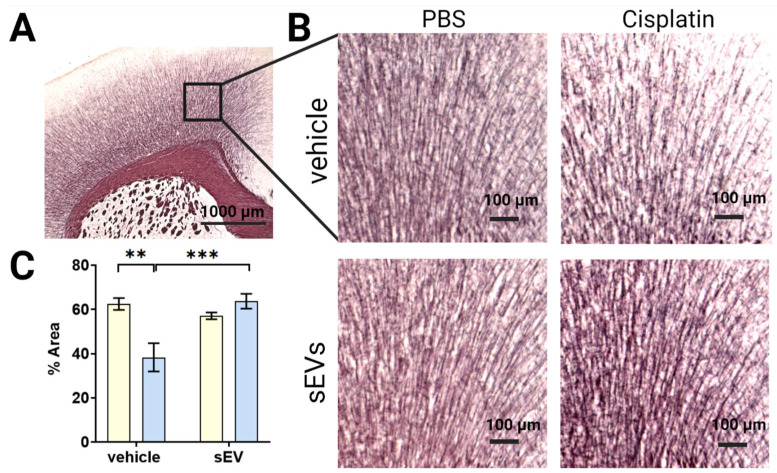
Intranasal administration of sEVs reverses cisplatin-induced damage of white matter. Mice were treated with PBS or cisplatin, followed by intranasal administration of PBS or sEVs. Brains were collected approximately 3 weeks after sEV administration, fixed, and cryopreserved. (**A**) Representative image showing Black-Gold II staining for myelin in the cingulate cortex. Position of area taken for quantification is outlined in black. Scale bar—1000 µm. (**B**) Representative images of cingulate cortex used for quantification in animals treated with PBS or cisplatin after sEVs or vehicle treatment. Scale bar = 100 µm. (**C**) Average % area stained with Black-Gold II (*n* = 4M/2F), quantification was performed using Image J software (https://imagej.nih.gov/ij/). Data were analyzed with two-way ANOVA for cisplatin and sEVs with Tukey’s post-hoc analysis, and results are expressed as mean ± SEM. ** *p* < 0.01, *** *p* < 0.001.

We previously published that cisplatin-induced cognitive deficits are associated with damage to the myelin in the cingulate cortex [13,14,17,22]. To investigate whether sEVs repaired the cisplatin-induced white matter damage, we visualized myelinated fibers in the cingulate cortex using Black Gold II staining. The results show that cisplatin significantly reduced the area staining positive for myelin in both males and females, indicating a loss of myelination (Figure 4C). Intranasal sEV treatment normalized the area staining positive for Black-Gold II, indicating restoration of myelin integrity.

### 2.5. Intranasally Administered sEVs Restore Synaptic Integrity in the Hippocampus of Cisplatin-Treated Animals

**Figure 5:**
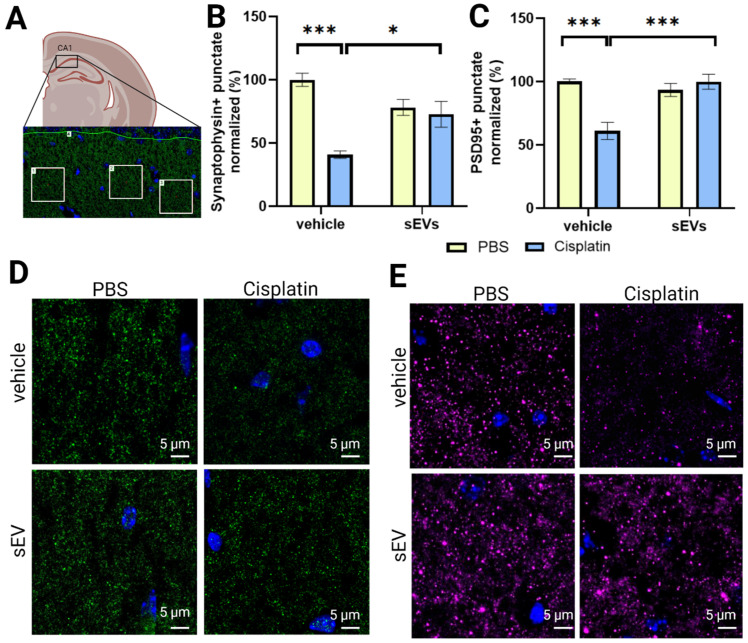
Expression of synaptic markers synaptophysin and PSD95 in the hippocampus of cisplatin- and cisplatin/sEVs-treated mice. Mice were treated with PBS or cisplatin, followed by nasal administration of PBS or sEVs. Brains were collected approximately 3 weeks after sEV administration, fixed, cryopreserved, and frozen. (**A**) Representative image showing synaptophysin staining in the mouse hippocampus. Position of area taken for quantification is outlined in black (numbered 4). Representative image of ROIs selected for quantification of synaptic punctate (outlined in white, numbered 1–3). Quantification of synaptophysin+ (**B**) and PSD95+ punctate (**C**) in animals treated with PBS or cisplatin after sEVs or vehicle administration (*n* = 4M/4F). Data were analyzed with two-way ANOVA for cisplatin and sEVs with Tukey’s post-hoc analysis, and the results are expressed as mean ± SEM. * *p* < 0.05, *** *p* < 0.001. Representative images of synaptophysin+ (**D**) and PSD95+ (**E**) synaptic punctate in animals treated with PBS or cisplatin after sEV or vehicle administration. Scale bar—5 µm.

Cisplatin treatment reduced the number of punctates staining positive for the presynaptic marker synaptophysin and the postsynaptic marker PSD95 in the CA1 region of the hippocampus [12,13]. Intranasal administration of sEVs normalized the number of punctates staining positive for synaptophysin or PSD95 in the CA1 of the hippocampus, indicating that both pre- and postsynaptic integrity was restored by intranasal sEV treatment (Figure 5).

### 2.6. Intranasally Administered sEVs Restore Morphology of Mitochondria

**Figure 6:**
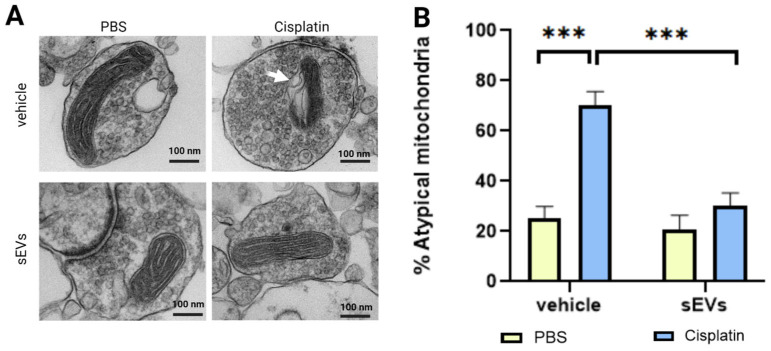
Morphology assessment of synaptic mitochondria in cisplatin- and cisplatin/sEV- treated mice. Mice were treated with PBS or cisplatin, followed by intranasal administration of PBS or sEVs. Hemibrains were collected approximately 3 weeks after sEV administration. Synaptosomes were isolated and fixed in 3% glutaraldehyde/2% paraformaldehyde. (**A**) Representative TEM images of synaptosomal mitochondria isolated from animals treated with PBS or cisplatin after sEV or vehicle administration. Arrow shows disrupted membrane and cristae after cisplatin treatment. Scale bar—100 nm. (**B**) Quantification of atypical mitochondria in animals treated with PBS or cisplatin after sEV or vehicle administration (*n* = 4M/4F). Data (10 images per animal) were analyzed with two-way ANOVA for cisplatin and sEVs with Tukey’s post-hoc analysis, and the results are expressed as mean ± SEM. *** *p* < 0.001.

We showed previously that cisplatin causes structural damage to synaptic mitochondria [13,17,35]. TEM analysis confirmed that cisplatin treatment causes damage to mitochondria of brain synaptosomes (Figure 6A, Supplementary Figure S6). Cisplatin causes mitochondrial swelling, membrane ruffling, and cristae disorganization (Figure 6A, arrow). Quantification of the percentage of these atypical mitochondria showed that intranasal administration of sEVs normalized mitochondrial morphology and reduced the % of atypical mitochondria to levels of the control group.

### 2.7. RNA Seq Analysis of the Hippocampus

**Figure 7:**
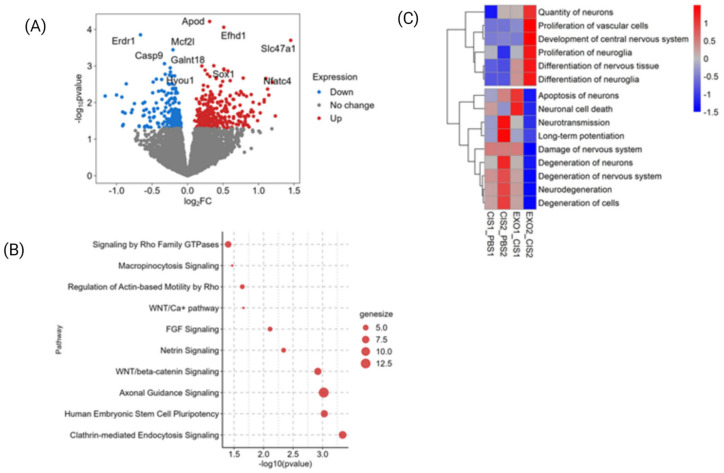
RNA seq analysis of the transcriptome in the hippocampus after cisplatin and sEV treatment. (**A**) Differentially expressed genes in the hippocampi of mice treated with cisplatin/sEV; genes showing significant difference in expression (*p* > 0.05, fold1) are labelled. (**B**) List of pathways upregulated in hippocampus after sEV treatment. (**C**) Functional enrichment analysis.

Our data show that two intranasal administrations of sEVs normalized cognitive impairment and brain white matter damage of cisplatin-treated mice. To get more insight into pathways activated after sEV administration in cisplatin-treated mice, we performed RNA seq analysis of the hippocampus (Figure 7A). Differential gene expression was assessed by comparing data from animals treated with cisplatin and an additional one or two doses of sEV (labelled EXO1 and EXO2), to time-matched controls treated only with cisplatin (labelled CIS1 and CIS2) (Figure 7). We found that a total of 1804 genes were differentially expressed 24 h after the first sEV dose was given, and a total of 1573 genes were differentially expressed 24 h after both sEV doses. The differentially expressed genes were analyzed by ingenuity pathway analysis (IPA) to determine pathways and functional enrichment as a result of sEV treatment.

In cisplatin-treated animals, functional enrichment analysis showed increased function related to neuronal degeneration, apoptosis, and necrosis over time. This indicated that cisplatin treatment is inducing neurotoxicity in the hippocampus (Figure 7C, CIS2_PBS2). After two sEV administrations, neuronal development and differentiation- and proliferation-related functions were significantly enriched (Figure 7C, EXO2_CIS2). The neuronal degeneration and damage-related functions were downregulated, suggesting that sEV treatment of cisplatin-treated animals activates genes related to repair and growth of neurons in the hippocampal region (Figure 7C, EXO2_CIS2).

## 3. Discussion

Chemotherapy-induced cognitive impairment is a neurotoxic side effect of cancer treatment. There is an urgent need for a therapeutic strategy that restores cognitive impairment, thereby increasing quality of life for patients whose cancers have been successfully treated but still experience the neurotoxic consequences of chemotherapies.

We show here for the first time that intranasal administration of two doses of MSC-derived sEVs completely resolves cisplatin-induced cognitive impairment in mice. Executive function, as well as spatial and learning memory of cisplatin-treated mice, are restored within two weeks after two intranasal sEV doses (Figure 1, Supplementary Figures S2 and S3). Furthermore, we show that sEV administration reverses the underlying structural damage of the mitochondria and changes in myelin complexity and in hippocampal synaptic integrity (Figure 4, Figure 5 and Figure 6). Further investigation is needed to provide a comprehensive understanding of the dose-dependent effects of sEV administration or the relative efficacy of sEV administration compared to alternative treatments.

We have previously demonstrated that cisplatin-induced cognitive impairment in mice can be observed using different behavior-testing paradigms, including NOPRT, PBT, 5-CSRT, and Y-maze. Impaired executive functioning due to chemotherapy is especially common in patients treated for breast cancer, and spatial learning is also impaired [36,37,38,39,40]. We previously showed that a test battery consisting of PBT and NOPRT can assess the therapeutic effect of MSCs and MSC-derived mitochondria [12,13,22]. We used this approach to assess if MSC-derived sEVs reverse cognitive deficits in cisplatin-treated mice that are related to executive functioning and spatial and working memory.

A single sEV administration showed little effect on performance in the puzzle box test (Figure 2E), and it took two sEV doses to restore cognitive performance (Figure 2D,E). This is consistent with our previous findings showing that recovery from chemobrain needs two intranasal doses of MSCs [22,41].

Therapeutic effects of sEVs depend greatly on their ability to easily reach the target tissue. For reversing CICI, we administered sEVs via the intranasal route as an efficacious way to deliver therapeutics directly to the brain. Intranasal administration circumvents the need for therapeutics to cross the blood-brain barrier to reach the brain [42,43]. Despite the generally argued ability of sEVs to cross the blood-brain barrier, delivery of therapeutic EVs to the brain after i.v. or i.p. administration is hampered by the accumulation of sEVs in peripheral organs (liver, lungs, intestine) [33,44]. In this regard, direct delivery of sEV to the brain may provide clinically relevant benefits, including reducing potential off-target effects. Intranasal delivery also enables targeting more directly to the brain regions that are affected by the pathology [45,46,47].

Our data show that sEVs delivered intranasally reach the brain in 1 h and remain in the brain for 48h. The distribution pattern of sEVs is consistent with the generalized neurotoxic effect of cisplatin since the fluorescent DiD signal was distributed throughout the entire brain (Figure 3). In our earlier studies using intranasal administration of the parental MSC lines or isolated mitochondria from MSCs, we observed that MSCs or the mitochondria first reach the meningeal compartment, where we could observe them for more than 72 h [13,48]. However, we did not detect a signal of sEVs in the meninges after intranasal administration (Figure 3), but the sEVs penetrated into the brain. After intranasal administration, particle size is crucial for therapeutic delivery beyond the olfactory bulb, with small particles in the size range of sEV (100 nm) being able to penetrate the brain directly [49].

Our RNA sequencing analysis pointed to upregulation of clathrin-mediated endocytosis, as well as micropinocytosis, which may be related to sEV uptake (Figure 7B). Clathrin-mediated endocytosis is a well-described uptake mechanism for vesicles up to 200 nm in size [50], which is consistent with the ZetaView data showing that most of the sEVs are between 80–120 nm in diameter. It has been shown that both neurons and glia take up sEV by clathrin-mediated endocytosis [51,52]. In contrast, macropinocitosis is not limited in size, and vesicles larger than 200 nm can be taken up [53,54,55]. Microglia selectively uptake sEVs from oligodendrocytes by macropinocytosis by engaging surface phosphatidyl serine (PS) [56]. This is interesting since cisplatin causes phosphoserine expression on the membranes of cells, which may underscore the importance of microglia in EV-mediated recovery [57].

Wnt signaling, netrin signaling, and axonal guidance signaling, which are upregulated after two sEVs doses, have previously been related to cognitive function [58,59]. In addition, netrin expression in the hippocampus plays a critical role in the regulation of spatial memory formation [60]. Our data also point to the role of the Nfatc4 gene, which is upregulated after sEV administration and involved in regulation of spatial memory (Figure 7A) [61]. While these data clearly point to the regenerative potential of MSC-derived sEVs, additional research is required to identify the biologically active components of sEV, as well as the key factors in reproducible production of biologically active sEVs.

In conclusion, we show that two doses of MSC-derived sEVs given intranasally reverse cisplatin-induced cognitive impairment and resolve structural damage. Our data strongly suggest that clinical translation of intranasal administration of MSC-derived sEVs for the treatment of cancer patients with chemobrain is possible in the near future. To fully elucidate the therapeutic potential of MSC-derived sEVs, future studies are needed to identify the active components of sEVs and their precise mechanism of action. Further comparative assessment of the effects of related biological therapies, including MSC and MSC-derived mitochondria, as well as assessing the use of sEVs for chemobrain as a result of other chemotherapeutics, is needed as well.

## 4. Materials and Methods

### 4.1. Preparation and Characterization of sEV from hBM MSC

Human bone marrow-derived MSC (p5) were cultured in complete culture medium (CCM) containing Minimal Eagle Essential Medium (MEM) (Corning Life Sciences, Corning, Painted Post, NY, USA) supplemented with GlutaMAXTM (Gibco, Life Technologies Corporation, Grand Island, NY, USA), 2 USP units/mL heparin (Fresenius Kabi USA, Lake Zurich, IL, USA), 7.5% heat inactivated Fetal Bovine Serum (FBS, Sigma-Aldrich, St. Louis, MO, USA), 2.5% platelet lysate (PLTMax^®^, Mill Creek Life Sciences, Rochester, MN, USA), and 1% penicillin-streptomycin (Sigma-Aldrich, St. Louis, MO, USA). When cells reached 80% confluency, the media were replaced with sEV harvesting media containing 10% exosome-depleted FBS (Life Technologies Corporation, Grand Island, NY, USA) for 48 h.

sEVs were collected using differential ultracentrifugation according to Thery et al. (2006) [62]. In short, culture supernatant was first centrifuged at 2000 and 10,000× *g* to remove dead cells and debris. The supernatant was then centrifuged for 70 min at 100,000× *g* using Optima XPN ultracentrifuge (Beckman Coulter, Pasadena, CA, USA) in an 70Ti rotor (k = 156). The resulting pellet was resuspended in PBS (Ca/Mg free) and centrifuged again under the same conditions. The sEV fraction was stored at −80 °C.

sEVs were characterized in accordance with the ISEV criteria [63]. Size distribution was assessed using ZetaView TWINN (Particle Metrix, Inning am Ammersee, Germany). The sample was diluted in particle-free Mili-Q water, and the recording was taken in scatter or fluorescent mode according to the manufacturers’ instructions.

For the Western blot, samples were prepared in reducing Laemmli buffer and separated on a 10% PAA gel. Gels were calibrated using Thermo Scientific™ PageRuler™ Prestained Protein Ladder. Proteins were transferred to a nitrocellulose membrane (Thermo Scientific, Rockford, IL, USA), and the membrane was probed using an anti-CD63 antibody (ab59479; Abcam, Waltham, MA, USA) and an anti-GM130 antibody (11308-1-AP, Proteintech, Rosemont, IL, USA).

sEVs were prepared for TEM by fixing in 2% paraformaldehyde, electron microscopy grade (Electron Microscopy Sciences, Hatfield, PA, USA). sEVs were deposited on formvar-coated 200 mesh copper grid (Electron Microscopy Sciences, Hatfield, PA, USA) stained with uranyl acetate and lead citrate and examined in a JEM 1010 transmission electron microscope (JEOL, USA, Inc., Peabody, MA, USA) at an accelerating voltage of 80 kV. Digital images were obtained using the AMT Imaging System (Advanced Microscopy Techniques Corp, Danvers, MA, USA).

### 4.2. Mice

8–10-week-old male and female C57BL/6J mice were procured from Jackson Laboratory (Bar Harbor, ME, USA). All animal experiments were performed at The University of Texas MD Anderson Cancer Center, Houston, Texas, according to procedures approved by the Institutional Animal Care and Use Committee. Mice were housed on a reversed 12 h dark/light cycle and had free access to water and food and were randomly assigned to treatment groups. Investigators performing behavioral testing and structural analysis were blinded for treatment.

### 4.3. Cisplatin Treatment and Nasal sEVs Administration

Mice received cisplatin (Accord Healthcare Inc, Durham, NC, USA) intraperitoneally (i.p.) in 2 rounds of 5 daily doses of 2.3 mg/kg in PBS with 5 days of rest in between, as previously published [13,14,17]. Control mice were injected with PBS. sEVs were given nasally 48 and 96 h after the last cisplatin dose. Before nasal sEVs administration, mice received hyaluronidase (20 U/mL) (Sigma Aldrich, St. Louis, MO, USA), 3 µL per nostril, to permeabilize the nasal mucosa, as published previously [13,22,48]. At 30 min after the hyaluronidase treatment, mice received 0.05 billion EVs/3 µL per nostril. Total EV dose was 0.1 billion. The control group received 3 µL of PBS per nostril in the same manner.

### 4.4. Cognitive Behavioral Testing

Behavioral testing to assess cognitive function was started 2 weeks after sEV treatment using the puzzle box test (PBT) for executive functioning and the novel object place recognition test (NOPRT) for short-term and spatial memory [13,19,22].

The PBT is performed in a standardized testing box, consisting of a bright compartment (55 cm × 28 cm) and a dark compartment (15 cm × 28 cm) connected by a tunnel (4 cm × 2.5 cm). Each mouse was placed individually in the bright compartment, and the time taken to enter the dark compartment was recorded. The tunnel was open (“easy” trial), covered with bedding (“intermediate”), or covered with a lid the same color as the base of the box (“difficult” trial). All trials are conducted on consecutive days in the following order: Day 1—three “easy” trials, Day 2—one “easy” and two “intermediate” trials, Day 3—one “intermediate” and two “difficult” trials, Day 4—two “difficult” trials.

For the NOPRT, each mouse was trained in a standardized 47 × 22 cm arena containing two identical objects for 5 min (“training” phase), returned to its home cage for 60 min, and reintroduced to the arena containing one now familiar object and a novel object in a new location. Time exploring the familiar and novel object was quantified using EthoVision XT 10.1 tracking software (Noldus Information Technology Inc., Leesburg, VA, USA). The discrimination index was calculated as—(T_Novel_ − T_Familiar_)/(T_Novel_ + T_Familiar_).

### 4.5. sEV Labelling and Tracking in the Brain

sEVs were incubated with the fluorescent dye DiD (1,1′-dioctadecyl-3,3,3′,3′- tetramethylindodicarbocyanine, 4-chlorobenzenesulfonate salt (Thermo Fisher, Grand Island, NY, USA)), as prescribed previously [31]. In short, sEVs were incubated with 1 µM DiD at 37 °C for 30 min. The sample was centrifuged for 70 min at 100,000× *g* using an Optima XPN ultracentrifuge (Beckman Coulter, Pasadena, CA, USA) in the 70Ti rotor (k = 156) to remove the excess dye. The resulting pellet was resuspended in PBS (Ca/Mg free). A total of 2 billion labelled sEVs in 2 doses of 3 µL per nostril were administered intranasally after pretreatment with hyaluronidase as described above.

Mice were sacrificed by CO_2_ exposure and perfused with ice cold PBS supplemented with 5 U/mL sodium heparin, and brains were collected and imaged in an IVIS Lumina (Perkin Elmer, Waltham, MA, USA) using the 640/700 nm excitation/emission filters. Intact brains were imaged from the axial/horizontal plane. Coronal images were obtained on 1.0 mm slices prepared using the mouse slicing matrix (Zivic catalog #BSMAS001-1) across hippocampal regions corresponding to section 19 of the C57BL/6J Atlas (−1.28 mm from bregma) (http://www.mbl.org/atlas170/atlas170_frame.html, accessed on 5 April 2021).

### 4.6. Myelin Integrity

Brains were collected after perfusion with PBS and fixed in 4% paraformaldehyde for 48 h and frozen in OCT (Sakura Fintek, Torrance, CA, USA). Coronal sections sized 25 µm were sliced using a Leica SM2010 R sliding microtome (Leica Biosystems Inc., Buffalo Grove, IL, USA). The sections were mounted and stained for myelin using the Black-Gold II kit (Biosensis, Thebarton, Australia) according to manufacturer’s instructions. Imaging was performed using an EVOS^®^ FL microscope (Thermo Fisher Scientific, AMG, Mill Creek, WA, USA), using a 4× and 20× objective. The percentage of the stained area was quantified using ImageJ with the Orientation J plugin (https://imagej.nih.gov/ij/, accessed on 15 February 2021).

### 4.7. Immunofluorecence Analysis

Mice were sacrificed by CO_2_ and perfused with ice-cold PBS supplemented with 5 U/mL sodium heparin and brains were fixed in 4% paraformaldehyde for 48 h, cryoprotected in sucrose, and frozen in an optimal cutting temperature compound (O.C.T., Sakura Finetek, Torrance, CA, USA). Coronal sections of 8 µm were cut with Leica CM3050S Cryostat (Wetzlar, Germany), mounted onto slides, post-fixed in 4% PFA for 1 h, washed in PBS, and blocked in 3% BSA, 5% normal goat serum (NGS), and 0.3% Triton-X in PBS for 2 h at room temperature. Sections were stained with rabbit anti-mouse synaptophysin (1:1000, AB 9272, EMD Millipore, Burlington, MA, USA) or rabbit anti-mouse PSD95 (1:1000, ab18258, Cambridge, UK) overnight at 4 °C, followed by Alexa 647 goat anti-rabbit (1:500, # A-21245, Thermo Fisher Scientific, Waltham, MA, USA) for 2 h at room temperature. All antibodies were diluted in antibody buffer (2% BSA, 2% NGS and 0.1% saponin in PBS). Nuclei were visualized with DAPI, and sections were mounted using FluorSaveTM (MilliporeSigma, Burlington, MA, USA).

For quantification, three regions of interest (ROIs) in the CA1 region of the hippocampus were imaged using a Nikon A1R Confocal Microscope (Nikon Instruments Inc., Melville, NY, USA) using 40× objective. The number of punctates larger than 0.38 µm in diameter per ROI were quantified using Nikon NIS-Elements Advanced Research (Nikon Instruments Inc., Melville, NY, USA) (https://www.microscope.healthcare.nikon.com/products/software/nis-elements, accessed on 29 June 2023).

### 4.8. TEM of Brain Synaptosome Mitochondria

Synaptosomes were isolated from hemibrains collected after PBS perfusion. Hemibrains were homogenized in a Dounce homogenizer with Syn-PER Synaptic Extraction Buffer (Life Technologies Corporation, Grand Island, NY, USA) and supplemented with Halt protease inhibitors (Thermo Scientific, Grand Island, NY, USA). The homogenates were centrifuged at 1000× *g* for 10 min at 4 °C, and the supernatant was diluted 1:1 with 1.3 M sucrose HEPES buffer and centrifuged at 20,000× *g* for 30 min at 4 °C.

The pellet containing synaptosomes was fixed with electron microscopy grade 3% glutaraldehyde/2% paraformaldehyde in 0.1 M cacodylate buffer, pH 7.3, post-fixed with 1% buffered osmium, and stained en bloc with 1% Millipore-filtered uranyl acetate. The samples were dehydrated in increasing concentrations of ethanol, infiltrated, and embedded in LX-112 medium, followed by polymerization in a 60 °C oven for approximately 3 days. Ultrathin sections were cut in a Leica Ultracut microtome (Leica, Deerfield, IL, USA), stained with uranyl acetate and lead citrate in a Leica EM Stainer, and examined in a JEM 1010 transmission electron microscope (JEOL, USA, Inc., Peabody, MA, USA) at an accelerating voltage of 80 kV. Digital images were obtained using an AMT Imaging System (Advanced Microscopy Techniques Corp, Danvers, MA, USA).

Qualitative assessment was conducted as published previously [13,17]. In short, mitochondria contained within synaptic vesicles were examined for swelling, cristae disorganization, and membrane ruffling. Mitochondria exhibiting one or more of these characteristics were labelled as atypical. Images (10 per mouse, 4 mice per group) were scored by two independent researchers blinded to treatment.

### 4.9. RNA Sequencing of the Hippocampus and Data Analysis

Animals were sacrificed by CO_2_ and perfused with ice-cold PBS supplemented with 5 U/mL sodium heparin 24 h after both sEV doses were given. The hippocampi were collected immediately and frozen in liquid nitrogen. Total RNA was extracted using RNeasy MinElute Cleanup Kit (Qiagen, Hilden, Germany). RNA sequencing was performed by Novogene Corporation Inc (Sacramento, CA, USA) on the NovaSeq PE150 System (Illumina, San Diego, CA, USA). The mRNA library was constructed by poly A enrichment, and 20 million raw pair reads were generated per sample.

The raw reads were obtained in the FASTQ format and used for the analysis. The FASTQC tool was used to check the quality of the raw reads [64]. To align the raw reads with reference mouse genomes (mm10), the STAR package was used [65]. From the alignment, gene counts were estimated for each sample using the featureCounts program from the Subread tool [66]. For the differential gene expression analysis between groups, we used the DEseq2 program [67]. The gene counts were normalized, and differential gene expressions were calculated for the comparisons of cisplatin- vs. PBS-treated and cisplatin/EVs vs. cisplatin-treated in each time point. For all the analysis, the *p*-value < 0.05 was used as a cutoff to select the differentially expressed genes. The pathway enrichment analysis was performed using the Ingenuity Pathway Analysis tool (IPA; Qiagen Inc., Hilden, Germany).

### 4.10. Statistical Analysis

Data were analyzed using GraphPad Prism version 8.0.0 for Windows (GraphPad Software, San Diego, CA, USA). Two or three-way ANOVA followed by two-tailed Tukey’s post hoc analysis was performed as appropriate. *p* < 0.05 was considered statistically significant.

## Supplementary Materials

The following supporting information can be downloaded at https://www.mdpi.com/article/10.3390/ijms241411862/s1.
