# Supplementary material for: Intranasally Administered MSC-Derived Extracellular Vesicles Reverse Cisplatin-Induced Cognitive Impairment
Source: Int J Mol Sci. 2023 Jul 24;24(14):11862. doi: 10.3390/ijms241411862 (PMC10380450; doi:10.3390/ijms241411862)
Supplement: Supplementary file 1 [file ijms-24-11862-s001.zip › ijms-2506752-supplementary.pdf]

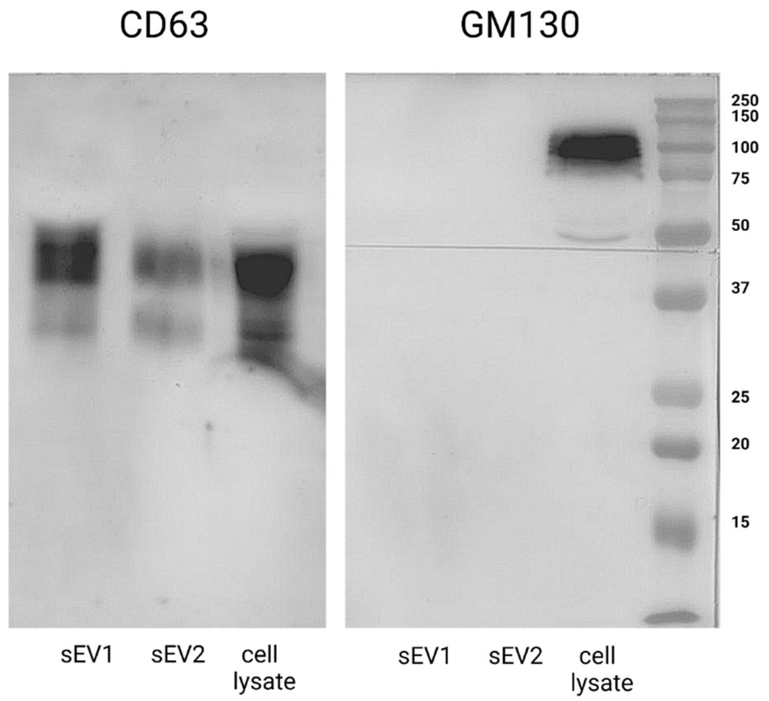

Supplementary Figure S1. Protein marker profile of BM MSC-derived sEV. sEV we probed for CD63 and anti-GM130 immunoreactivity in western blot. hMSC lysate was used as control.

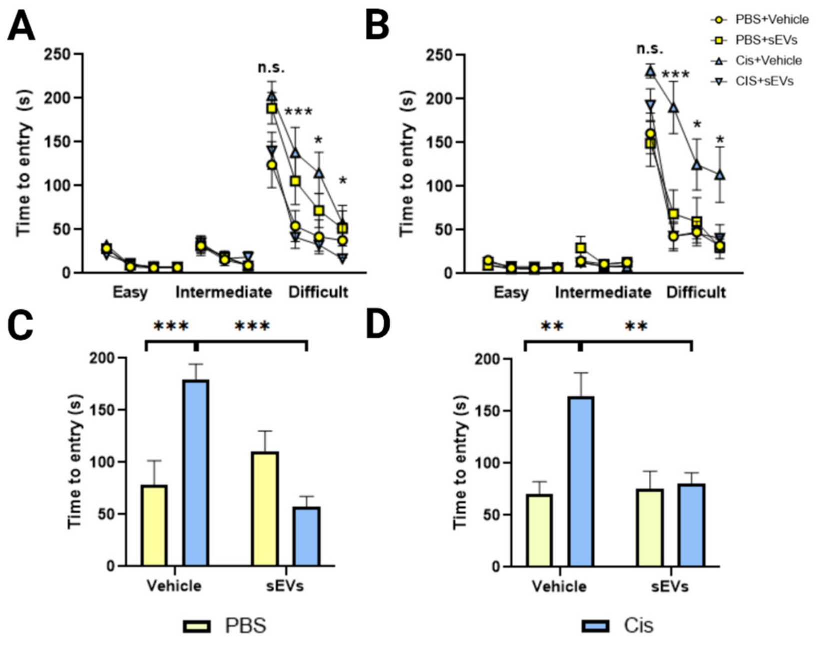

Supplementary Figure S2. Cisplatin and sEV treatment have the same effect in male and female mice in PBT. There is no effect on animal performance in PBT easy and intermediate trials, in both males (n=12) **(A)** and females (n=8) **(B)**. Average time to entry into the dark chamber recorded in 4 difficult trials in males (n=12) **(C)** and females (n=8) **(D).** Data were analyzed with two-way ANOVA, for cisplatin and sEV with Tukey's post-hoc analysis and the results are expressed as mean ± SEM. ***p<0.001, **p<0.01, *p<0.1.

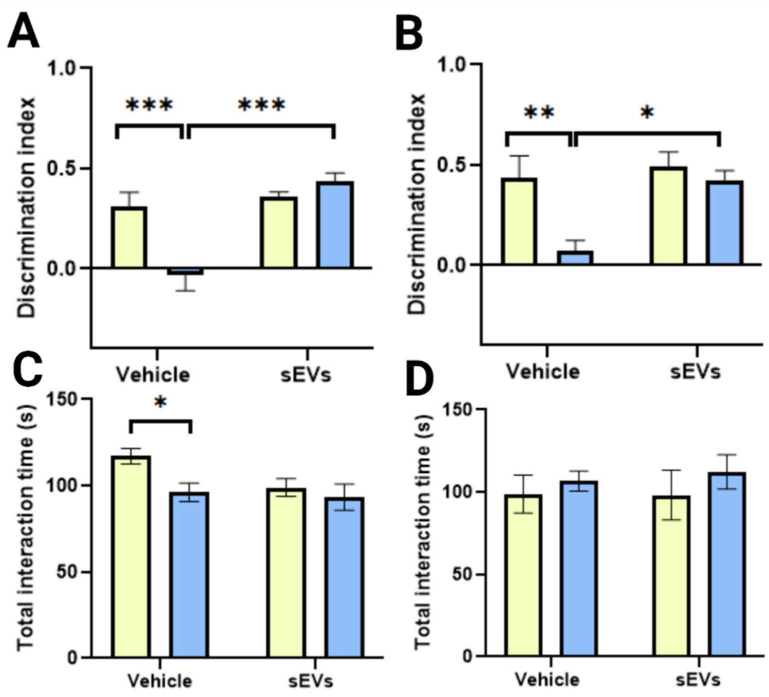

Supplementary Figure S3. Cisplatin and sEV treatment have the same effect in male and female mice in NOPRT. sEV treatment restores DI in males (n=12) **(A)** and females (n=8) **(B).** Cisplatin and sEV effect on total interaction in NOPRT in (n=12) **(C)** and females (n=8) **(D).** Data were analyzed with two-way ANOVA, for cisplatin and sEV with Tukey's post-hoc analysis and the results are expressed as mean ± SEM. ***p<0.001, **p<0.01, *p<0.1.

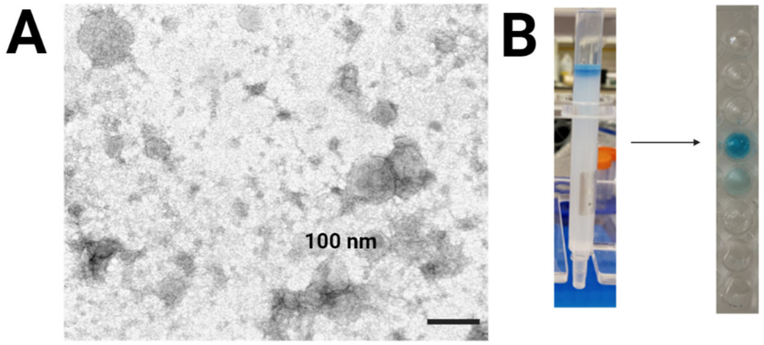

Supplementary Figure S4. TEM of DiD-stained sEV. (A) Typical morphology of sEV is preserved during staining. **(B)** Size exclusion chromatography showed DiD was present only in the exosome fraction.

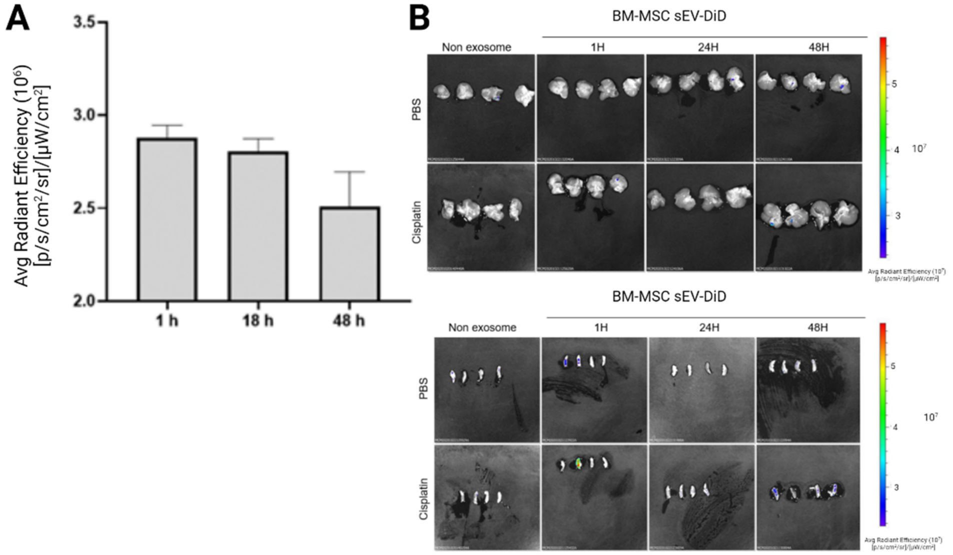

Supplementary Figure S5. Quantification of DiD-signal in the brain and spatiotemporal distribution of signal in peripheral tissues. (A) The fluorescent signal was quantified using LivingImage software. Data were analyzed with

student t-test. No significant difference was found. **(B)** No fluorescent signal was detected in liver and spleen.

A

B

C

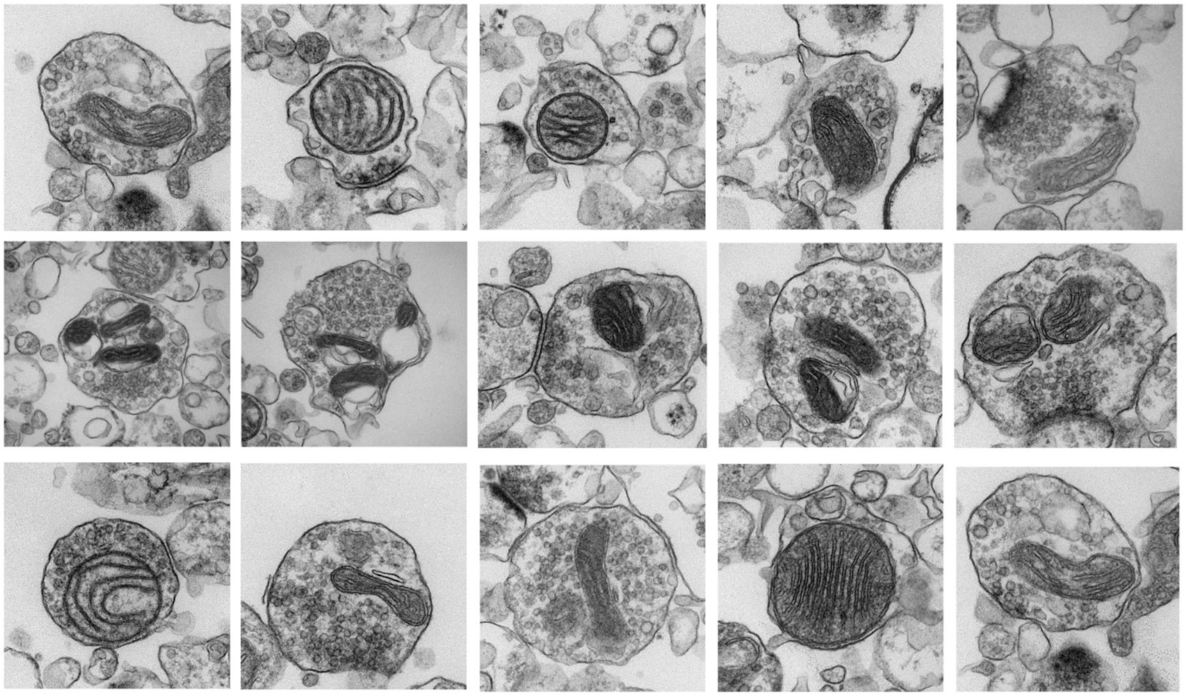

Supplementary Figure S6. Representative images of mitochondrial morphology. (**A**) Animals treated with PBS (**B**) Animals treated with cisplatin (**C**) Animals treated with cisplatin/sEV.
